# Alcohol use in severely injured trauma patients

**DOI:** 10.1038/s41598-020-74753-y

**Published:** 2020-10-21

**Authors:** Antti Riuttanen, Saara J. Jäntti, Ville M. Mattila

**Affiliations:** 1 Department of Orthopaedics, Faculty of Medicine and Health Technology and Tampere University Hospital, Tampere University, Tampere, Finland; 2 School of Medicine, University of Tampere, Tampere, Finland

**Keywords:** Health care, Fracture repair, Risk factors, Trauma

## Abstract

Alcohol is a major risk factor for several types of injuries, and it is associated with almost all types and mechanisms of injury. The focus of the study was to evaluate alcohol use in severely injured trauma patients with New Injury Severity Score (NISS) of 16 or over, and to compare mortality, injury severity scores and mechanisms and patterns of injury between patients with positive and negative blood alcohol levels (BAL). Medical histories of all severely injured trauma patients (n = 347 patients) enrolled prospectively in Trauma Register of Tampere University Hospital (TAUH) between January 2016 to December 2017 were evaluated for alcohol/substance use, injury mechanism, mortality and length of stay in Intensive Care Unit (ICU). A total of 252 of 347 patients (72.6%) were tested for alcohol with either direct blood test (50.1%, 174/347), breathalyser (11.2%, 39/347), or both (11.2%, 39/347). After untested patients were excluded, 53.5% of adult patients (18–64 years), 20.5% of elderly patients (above 65 years) and 13.3% of paediatric patients (0–17 years) tested BAL positive. The mean measured BAL for the study population was 1.9 g/L. The incidence of injuries was elevated in the early evenings and the relative proportion of BAL positive patients was highest (67.7%) during the night. Injury severity scores (ISS or NISS) and length of stay in ICU were not adversely affected by alcohol use. Mortality was higher in patients with negative BAL (18.2% vs. 7.7%, p = 0.0019). Falls from stairs, and assaults were more common in patients with positive BAL (15.4% vs. 5.4% and 8.7% vs. 2.7%, p < 0.006, respectively). There were no notable differences in injury patterns between the two groups. Alcohol use among severely injured trauma patients is common. Injury mechanisms between patients with positive and negative BAL have differences, but alcohol use will not increase mortality or prolong length of stay in ICU. This study supports the previously reported findings that BAL is not a suitable marker to assess patient mortality in trauma setting.

## Introduction

Alcohol is an important contributor to the global burden of disease^1–3^. Indeed, unintentional and intentional injuries are the cause of almost half (46%) of the global burden in alcohol-related mortality^1^. Between 1990 and 2013, Finland had the highest burden of alcohol-attributed diseases in Scandinavia^4^. Moreover, accidents are the fourth most common cause of death in Finland^5^. The most common accidents are falls, falls from a height, poisonings, motor vehicle accidents, drownings, suffocations and fires^6^.

Alcohol is known to have a negative effect on health, and it is a major risk factor for several types of injuries^7–9^. An increased blood alcohol level (BAL positive) is associated with almost all types and mechanisms of injury, including falls, cycling accidents, motor vehicle crashes, head traumas and assaults^10–14^. A literature review from 2011 estimated that the weighted incidence of alcohol-related visits to American trauma centres could be 32.5%^15^.

Alcohol intake is linked with an increased incidence of head trauma. Furthermore, the severity of craniofacial injury has been shown to be higher in intoxicated patients^16^. Head trauma is commonly caused by assault, fall or cycling accident^13^. In cycling accidents, one third of the injured cyclists were reported to be BAL positive and head injuries were more common in this group^11^. Driving under the influence of alcohol increases the risk of fatal and non-fatal driving accidents. A possible explanation for this is that intoxicated drivers usually take more risks and drive faster. Further, they are also more likely to suffer serious injury or death compared with sober drivers^17,18^.

The association between alcohol intoxication and injury severity, mortality and length of stay (LOS) in hospital is, however, inconsistent in the literature. Indeed, the majority of studies have found no difference in mortality^19,20^ and length of hospital of stay^21,22^ between BAL positive and BAL negative patients. However, some studies have reported a higher mortality rate^21,23^ and longer length of hospital stay^23^ among BAL positive trauma patients. Based on the findings of some studies, BAL positive patients had higher injury severity scores (ISS) when examined for minor trauma (ISS less than 16)^24^, whereas in severely injured patients (ISS greater than or equal to 16) there was no difference in ISS between BAL positive and BAL negative patients^20,25^.

The primary objective of the present study was to evaluate alcohol use in severely injured trauma patients in three age groups (paediatric, working age and elderly patients). A secondary objective was to analyse and to compare the mechanisms and patterns of injury between BAL positive and BAL negative patients. A further aim was to investigate whether BAL positive had an influence on the outcome, as measured by one-year mortality and length of stay in Intensive Care Unit (ICU).

## Material and methods

### Setting

The study was conducted at Tampere University Hospital. Tampere University Hospital (TAUH) is located in the Pirkanmaa region of Finland with a population of approximately 500,000. TAUH serves as a tertiary and highly specialised trauma care unit for the surrounding 3 hospital districts and has a catchment area of approximately 900,000 inhabitants. TAUH provides 24-h in-house or immediate service in orthopaedic surgery, neurosurgery, anaesthesiology, emergency medicine, radiology, internal medicine, plastic surgery, oral and maxillofacial, paediatrics and critical care.

Approximately 150 to 200 severely injured patients with a minimum New Injury Severity Score (NISS) of 16 are treated annually at TAUH. All trauma patients treated at TAUH who meet the inclusion criteria (NISS 16 or over, minimum MAIS (Maximum Abbreviated Injury Score) 3, treated at ICU or High Dependency Unit (HDU) are enrolled prospectively into the TAUH Trauma Register, which was founded in 2015. Patients with fatal head injuries without viable prognosis defined by a neurosurgeon are excluded from the register.

### Inclusion criteria and patients

In this study, we examined the associations of positive blood alcohol concentration and drug use in multiple trauma patients (NISS 16 or over, minimum MAIS 3) based on injury severity, injury pattern, mechanisms of injury, mortality and ICU stay. All patients enrolled in the TAUH Trauma Register in 2016 and 2017 were included in the study, making a total of 347 admission with 345 unique patients (age range 4–93 years). Two of the patients had two separate, unrelated accidents during the 2-year study period.

The full medical histories of the patients were evaluated for injury mechanisms and the role of alcohol or narcotics at the moment of trauma. The data collected included the cause and type of injury, injury pattern, admission time, time of day the injury occurred, patients’ age and sex and the use of alcohol and narcotics. Patients were classified by age as paediatric (0–17 years), adults (18–64 years) and elderly (above 65 years), and the use of alcohol and drugs was evaluated in each group.

### Evaluation of alcohol and narcotics use

The use of alcohol was confirmed or excluded by either breathalyser test, direct blood alcohol level (mmol/L) or both. To evaluate mean blood alcohol levels direct blood alcohol levels were converted to g/L (1 g/L = 21.7 mmol/L). When either test was positive, the patient was labelled as “BAL positive”. Patients with negative results were labelled “BAL negative”. Patients who were not tested were labelled as “BAL untested” and were excluded from analysis.

The use of narcotics was evaluated by urine test for benzodiazepines, cocaine, amphetamine, cannabis and opiates. Furthermore, the use of narcotics was separately categorised as either positive or negative for each substance. When a substance was found in the urine test, the patient was classified as “narcotics positive”. As the rate of testing was low, “untested patients” were classified as “narcotics negative”.

### Mechanisms and pattern of injuries

The causes of injuries were classified into the ten most common injury types. Falls from a height included all falls from a roof, ladder, tree or scaffolding. When sufficient information was available to declare a fall intentional, it was classified as attempted suicide. Falls on the same level included all falls on the ground, falls on the floor and falls in the street. Assaults were classified as all physical abuse, shootings (2) and stabbings (6). Bicycle accidents included all injuries that occurred while riding a bicycle, such as falls, collisions and injuries during leisure activities. Motor vehicle accidents included all crashes and driving off the road. Pedestrian and cyclist motor vehicle accidents included crashes and collisions involving an unprotected pedestrian or cyclist with a car, truck or train. Other accidents included injury mechanisms and types that did not fit in the previous classifications. These accidents occurred fewer than five times and included crush injuries and injuries caused by falling items.

Injury patterns were based on the Abbreviated Injury Scale (AIS)^26^ with pelvic injuries classified as lower extremity injuries. Minor (AIS1) injuries, such as cuts and bruises, were excluded from the analysis. The severity of the injuries was classified according to AIS version 2015. Both the Injury Severity Score (ISS) and the New Injury Severity Scores (NISS) were calculated^27,28^.

### Mortality and care at ICU/HDU

The 1-year mortality rate and length of stay in ICU/HDU were obtained from the intensive care unit’s database (Intensium) at TAUH. Mortality data for five foreign nationals were not available and these patients were therefore excluded from the mortality analysis.

### Statistics

All data were collected in Microsoft Excel and statistical analysis was performed with R Studio 4.0.0 with plotrix, ggplot2, epitools and gmodels extra packages. We assumed no homogeneity between patients with positive and negative BAL. The *x*^2^, Welch t-test and Mann–Whitney test were used. A p-value less than 0.05 was considered statistically significant.

### Ethics approval and consent to participate

Under Finnish legislation, the current study is exempt from the need to obtain ethical approval because of its retrospective nature, as stated by Regional Ethics Committee of the Expert Responsibility area of Tampere University Hospital.

## Results

### Population

Patient characteristics are presented in Table 1. In total, 76.2% (192/252) of the study population were male and 23.8% (60/252) were female. The mean age of the patients was 50.7 (SD 20.4), ranging from 12 to 93 years. Median ISS was 24 (range between first quartile (16) and third quartile (26)) and NISS 29 (Q1 24, Q3 38) for the whole study population. One patient with NISS of 75 had 3 separate AIS 5 injuries. BAL positive patients had lower ISS and NISS than BAL negative patients. Moreover, 15 patients were classified as paediatric, 159 as adults and 78 as elderly. Patient characteristics according to age are presented in Table 2.

**Table 1:** Patient characteristics. Severely injured patients, Tampere University Hospital, Finland, 2016–2017, n = 252.

	Alcohol involved		Alcohol not involved		p-value
	n = 104	%	n = 148	%	
**Gender**					0.083
Male	85	81.7	107	72.3	
Female	19	18.3	41	27.7	
**Age**					< 0.001
0–17	2	1.9	13	8.8	
18–64	85	81.7	74	50.0	
65-	17	16.3	61	41.2	
ISS*, median (Q_1_-Q_3_)	19.5	(13.75—25)	25	(18—26)	< 0.001
NISS† , median (Q_1_-Q_3_)	27	(21.75—34)	29	(24—38.75)	0.005
ICU‡ days, median (Q1—Q3)	2	(1–6)	2	(1–5.25)	0.25
**At least one injury in AIS§ region (min. AIS 2)**					
Head	77	74	116	78.4	0.42
Neck	10	9.6	20	13.5	0.75
Face	1	1.0	0	0	0.41
Thorax	27	26	44	29.7	0.51
Abdomen	8	7.7	14	9.5	0.62
Spine	28	26.9	40	27	0.99
Upper extremity	15	14.4	30	20.3	0.23
Lower extremity	6	5.8	17	11.5	0.12
Pelvis	5	4.8	14	9.5	0.17

*Injury Severity Score.

^†^New Injury Severity Score.

^‡^Intensive Care Unit.

^§^Abbreviated Injury Scale.

**Table 2:** Patient characteristics according to age.

	Age 0–17	Age 18–64	Age 65-	p-value
	n = 15	n = 159	n = 78	
**Gender**				
Male	13 (86.7)	124 (78)	55 (70.5)	0.28
Female	2 (13.3)	35 (22)	23 (29.5)	
ISS*, median (Q_1_-Q_3_)	24 (20.5—29)	22 (16–25)	25 (17–25.75)	0.18
NISS†, median (Q_1_-Q_3_)	34 (24 -36)	29 (22–37)	29 (25–38)	0.64
BAL‡				
BAL +	2 (13.3)	85 (53.5)	17 (21.8)	< 0.001
BAL-	13 (86.7)	74 (46.5)	61 (78.2)	
ICU§ days, median (Q1—Q3)	2 (1–4)	2 (1–5.5)	3 (1–6)	0.069
**At least one injury in AIS¶ region (min. AIS 2)**				
Head	12 (80)	115 (72)	66 (85)	0.10
Neck	2 (0)	23 (14)	5 (6)	0.20
Face	0 (0)	0 (0)	1 (1)	0.33
Thorax	9 (60)	51 (32)	11 (14)	< 0.001
Abdomen	2 (13)	17 (11)	3 (4)	0.17
Spine	6 (40)	39 (25)	23 (29)	0.36
Upper extremity	7 (47)	29 (18)	9 (12)	0.005
Lower extremity	3 (20)	17 (11)	3 (4)	0.070
Pelvis	2 (13)	12 (8)	5 (6)	0.65

*Injury Severity Score.

^†^New Injury Severity Score.

^‡^Blood Alcohol Level.

^§^Intensive Care Unit.

^¶^Abbreviated Injury Scale.

### Alcohol and narcotics use

A total of 252 of 347 patients (72.6%) were tested for alcohol with either direct blood test (50.1%, 174/347), breathalyser (11.2%, 39/347), or both (11.2%, 39/347). Moreover, 76.4% of adult patients, nearly half (46.9%) of paediatric patients and 72.9% of elderly patients were tested for alcohol use. After untested patients were excluded, 53.5% of adult patients (18–64 years), 20.5% of elderly patients (above 65 years) and 13.3% of paediatric patients (0–17 years) tested BAL positive. Mean BAL tested with breathalyser for the whole study population was 1.9 g/L (SD 0.9 g/L) or 44 mmol/L (SD 18.4 mmol/L). Twenty-three patients (6.6%) were tested for narcotics and 13 were positive. Positive tests included benzodiazepines (11), cocaine (2), amphetamine (5), cannabis (7) and opiates (11). All the positively tested patients were adults, and more than half were also BAL positive (8/13).

**Figure. 1:**
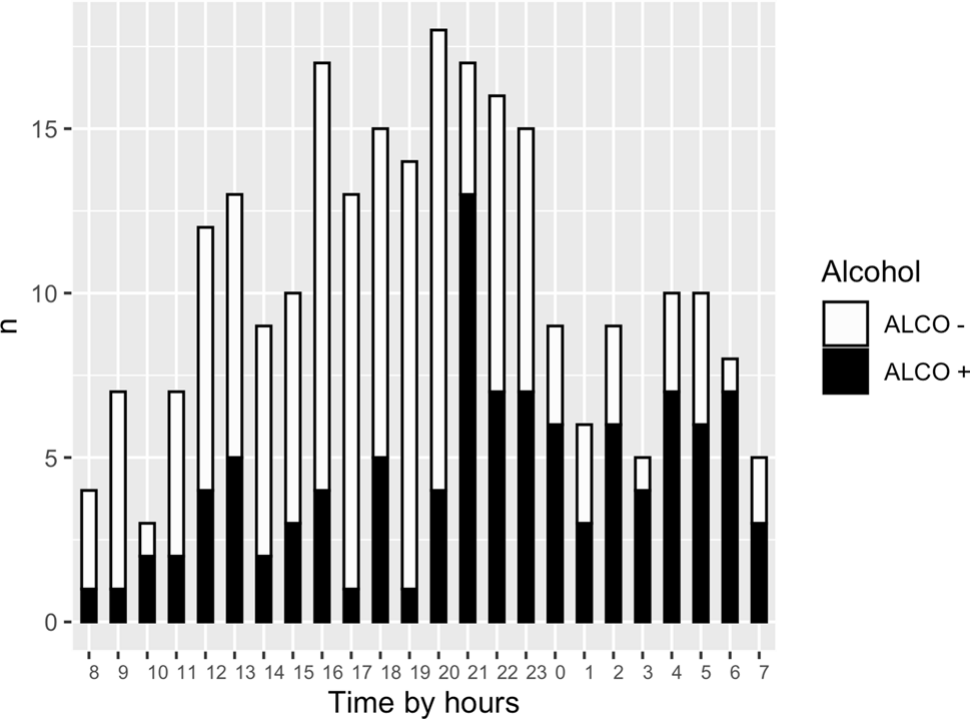
Distribution of injuries by time and relative rate of alcohol use. Alcohol involved = ALCO + . Alcohol uninvolved = ALCO−.

The relative rate of positive alcohol tests shows a clear pattern of increase towards the end of the day and night (Fig. 1). Approximately one in three patients tested BAL positive daytime (30.8%) and early evening (32.2%). However, during the night, the majority of trauma patients were BAL positive. After midnight, 67.7% of all patients and 76.6% (36/47) of adult patients were BAL positive. Moreover, patients admitted after midnight had a mean BAL of 1.9 g/L (SD 0.9 g/L). The highest measured BAL after midnight was 3.8 g/L or 84 mmol/L. The majority of these patients (86.1%) had BAL higher than 1.0 g/L.

### Injury pattern

The distribution of injuries according to the modified AIS classification is shown in Tables 1. There were no notable differences between patients with positive or negative BAL.

### Injury mechanism

The 10 most common injury mechanisms in descending order are presented in Table 3. Falls from stairs, and assaults were more common in patients with positive BAL (15.4% vs. 5.4% and 8.7% vs. 2.7%, p < 0.006, respectively). Motorcycle accidents and pedestrian auto or cyclist auto accident we less common in patients with positive BAL (2.8% vs. 8.8% and 1.9% vs. 6.1%, p < 0.006, respectively). There were 6 (2.4%) patients with penetrating trauma (2 shootings and 4 stabbings).

**Table 3:** Mechanisms of injury in descending order.

	Alcohol involved		Alcohol not involved	
	n = 104	%	n = 148	%
Falls on same level	39	37.5	46	31.1
Motor vehicle accidents	21	20.2	35	23.6
Falls from height (> 1 m)	6	5.8	14	9.5
Motorcycle accidents	3	2.8	13	8.8
Pedestrian or cyclist auto accidents	2	1.9	9	6.1
Falls from stairs	16	15.4	8	5.4
Assaults	9	8.7	4	2.7
Other/Miscellaneous	1	0.9	10	6.8
Biking accidents	3	2.9	5	3.4
Riding accidents	0	0	1	0.7
Attempted suicides	4	3.8	3	2.0

X2 = 24.64, df = 10, p < 0.006.

### Survival and length of stay in ICU

In total, 35 (13.9%) patients died within 1 year after trauma. Both patients who had two separate injury occasions were alive at 1 year. The 1-year mortality rate for BAL positive patients was 7.7% (8/104) and 15.5% (27/148) for BAL negative patients (p = 0.019). Patients who died during the first year were older (mean age 65.4) and had a higher NISS (median 37, range between first quartile (26) and third quartile (50)) than the rest of the population (mean age 48.3 years, median NISS 29, Q1 = 24, Q3 = 38) (p =  < 0.001 for age and NISS). Roughly half of the deaths (42.8%) occurred during first 30 days after trauma and majority (88.6%) during first 90 days after trauma. There were no deaths among BAL positive patients after 90 days. Thirty-day mortality rate for BAL positive patients was 4.8% and 12.8% in BAL negative patients.

All trauma patients were treated for a total of 1067 days in ICU. The median LOS in ICU for all trauma patients was 1.8 (Q1 = 0.9, Q3 = 4.7) The difference in LOS in ICU between patients with BAL positive or BAL negative was not statistically significant (p = 0.25).

## Discussion

Roughly half of the severely injured adult trauma patients (53.5%) treated at TAUH tested BAL positive with a mean BAL of 1.9 g/L. Alcohol use among severely injured elderly and paediatric populations was, however, less common as roughly 1 in 5 elderly (20.5%) patients and only 2 in 15 paediatric (13.3%) patients were BAL positive. Of the whole study population 41.2% of patients tested BAL positive (104/252). This finding is in line with previous studies which have shown that alcohol use among trauma patients treated at emergency departments is common. In a prospective Finnish study from 2005, Savola et al. reported that up to 51% of working age trauma population had alcohol in their blood on admission, and 86% of these had reached BAL of 1.0 g/L^13^. Recent retrospective study which extracted their data from American National Trauma Bank (NTDB, years 2007–2010) found out that roughly one third of trauma patients brought to level 1 or level 2 trauma centres tested positive for alcohol^22^. Furthermore, in 2013 Kowalenko et al. reported similar results from the same databank (NTDB, years 2000–2005) as 28% of trauma patients in register tested positive for alcohol^24^. In this study alcohol use was most prevalent in age groups between 21 and 50 years. Based on 15 studies a weighted average of prevalence of 32.5% alcohol-related visits to American trauma centres has been given^15^. However, the screening rate among these studies varied considerably from 30.7% to 98.6%, and used mainly retrospective data, with focus on urban level I trauma centres. In present study, the alcohol level was determined by direct blood test (69.0%, 174/252), breathalyser (15.5%, 39/252) or both (15.5%, 39/252). As most of the patients 84.5% (213/252) were tested with direct blood test or both tests, we had to rely on breath analyser result in only 15.5% (39/252) of the patients.

In our study, we noticed an interesting pattern in the incidence of injuries and in the relative rate of alcohol use during the course of the day. We found the incidence of injuries is relatively low in the morning, but then starts to increase and reaches its peak in the early evening**.** After that, it decreases during the night-time and towards the morning. However, at night, when the incidence of injuries starts to decline, the relative proportion of alcohol intoxicated patients starts to rise. During the night-time, over half of the trauma patients admitted have alcohol in their blood system, with the highest rate observed in the adult population (76.6%) Furthermore, as the majority of these patients (86.1%) had a BAL higher than 1.0 g/L, it is very likely that alcohol intoxication was the cause of the injury. The highest BAL of 3.8 g/L was measured after midnight. The highest overall rate of BAL positive patients admitted in ER was during the 8-h consecutive time period from 21:00 to 07:00 when 62.3% (65/107) of patients tested BAL positive. This is most likely associated with both general drinking habits and closing time of restaurants and bars in Finland.

In this study, we observed that the screening for narcotics use was scarce as only 6.6% of patients were tested. This is likely because substance screening at TAUH is done from the patient’s urine, making it more cumbersome than alcohol screening, which is done by either breathalyser or direct blood test. It may also be that substance screening is not done on a routine basis because historically substance use in Finland has been perceived to be less common than in the rest of Europe. However, it was noticed in recent wastewater studies that the use of illegal stimulant drugs in Finland is increasing^29–31^. Therefore, it is likely that substance use among trauma patients may also increase in future. The use of narcotics combined with alcohol was common as more than half of the positive narcotic test results came from the BAL positive group. However, due to the limited testing, the association between narcotics use and trauma injuries in Finland remains unclear.

This study supports earlier findings that among severely injured multiple trauma patient’s injury severity scores (ISS or NISS) or LOS in ICU are not adversely affected by alcohol use^21–25^. In our study, one-year mortality in patients with BAL positive was found to be lower than for the rest of the cohort. Concerning mortality, the literature is somewhat inconsistent. Kowalenko et al. reported increased mortality among BAL positive patients was also found in patients 40 years and older in another study^24^. In other age groups mortality was lower^24^. However, only 47% of patients in study were tested for alcohol and the researchers also didn’t have access to specific alcohol levels. Hadjizacharia et al. reported in retrospective match-pair analysis of 386 alcohol positive and alcohol negative patients almost twice as high as mortality (23% vs. 13%) in alcohol positive patients than in alcohol negative patients^21^. The observed mortality rate remained higher also when patients were stratified by ISS (< 16, 16–25 or > 25) However, the problem with ISS is that it only counts the most severe injury per body region as opposed to NISS which can count up to three injuries per body region. For example, a gunshot to abdomen, which could damage various organ together with large vessels could provide significantly different severity scores, depending on whether it was evaluated by ISS or NISS. As the rate of penetrating injuries in this study was high (22.3%) it is possible that the use of ISS as injury scoring system could have caused some bias in evaluation of mortality. Together with the notion that alcohol positive patients presented themselves with lower initial blood pressure and GCS, it is likely that alcohol positive patients were initially more critically ill than alcohol negative patients, and therefore more at risk of death. However, we should note that in our study the rate of penetrating injuries was scarce (only 2.4%, 6/252) and therefore the results may not be comparable. On the other hand, in significantly larger retrospective analysis with 92,959 propensity-matched trauma patient pairs no clinically significant difference in mortality rates between the two groups was seen^22^. Although the rate of screening alcohol in study is not known, the overall large quantity of patients in this study enabled more reliable and interesting statistical comparison between alcohol positive and negative patients.

Indeed, in our study, 1-year mortality in patients with BAL positive was found to be lower than for the rest of the cohort. This finding is probably due to the differences in trauma profile and higher injury severity scores among BAL negative patients. Based on patient medical histories, we observed two injury mechanisms (falling from stairs and assaults) that were overly represented in patients with positive BAL. Altogether, more than half of all accidents in this study were caused by some kind of fall 51.2% (105/252). Motorcycle accidents and pedestrian auto or cyclist auto accidents, which can all be considered as high energy traumas, were more common in patients with negative BAL. Injury patterns were however similar. The patients who died during the first year were older and mortality rose with increasing head injury AIS score. This supports the premise that old age and severe head trauma have more of an effect on mortality than alcohol use, even when alcohol use adds to the risks of head trauma. Almost half of the deaths (42.8%) occurred during first 30 days after injury and majority (88.6%) with-in 90 days after injury. It is debatable whether the 4 deaths (14.8%, 4/27) that occurred after 90 days in BAL negative group have association to injury. There were no deaths among BAL positive patients after 90 days.

An obvious limitation of this study is its retrospective nature. Alcohol and substance use were not systematically screened and in total 27.4% (95/347) of patients were not tested for alcohol and were therefore excluded from study. It is possible that this exclusion of untested patients may have caused some bias in results. Roughly half of “BAL untested” (46/95) patients were either paediatric (17) or elderly (29) patients, who are usually perceived less likely to be intoxicated than adult trauma patients. However, it is worth to remember that in 2013 in Finland the Parliamentary Ombudsman issued a decision stating that the health care unit has no right to systematically test all trauma patients with breathalyser as this would constitute interference with the patient’s personal integrity and privacy^32^. As it is, testing for alcohol in trauma patients cannot be a norm anymore in Finland. However, establishing whether or not the patient is under the influence of alcohol is often medically justified. Furthermore, in our present study testing for alcohol can still be considered relatively high. For example, in Kowalenko’s study from 2013 less than half (47%) of study population were tested for alcohol^22^.

It is possible that some selection bias may have occurred as result of TAUH’s Trauma register’s protocol of excluding patients with fatal head injuries without viable prognosis. These patients are usually institutionalized, geriatric patients who have sustained severe cerebral hematoma as a result of low-energy fall. Because injury severity scores are calculated on basis of anatomical classification these patients yield disproportionally high injury severity scores with-out representing true polytrauma patients. It is also evident that current study can be affected by not including the patients who died at the scene of the accident. We however know that the rate of alcohol intoxication (BAC > 0.5 g/L) among fatal traffic accidents in Finland is 25%^33^. As we compare that to the proportion of severely injured patients with positive BAL in this study, we see that the relative rates of alcohol intoxications are somewhat comparable.

The definitive strength of this study is the study population, which was a prospectively collected trauma cohort from the trauma registry of a single large hospital responsible for all major trauma care in its catchment area. In addition, all medical histories and laboratory tests were available to the investigators and all the relevant history was carefully inspected to accurately define injury mechanisms.

## Conclusions

In this retrospective study we found out that alcohol use among severely injured trauma patients in Finland is common. Roughly half of adult (aged 18–64 years) trauma patients (53.5%) were under the influence of alcohol at the time of the injury with mean BAL of 1.9 g/L. The highest prevalence of alcohol use was observed during night-time when 76.6% of adult patients had positive BAL. One-fifth of elderly (20.5%) and 2 in 15 (13.3%) of paediatric patients tested BAL positive. Of the whole study population 41.2% of patients tested BAL positive. More than half of injuries in BAL positive patients were caused by falling on the same level or falling from stairs. This is likely because high alcohol blood volume has negative effect on proprioception. Motorcycle accidents and pedestrian auto or cyclist auto accident, which can be considered as high energy trauma, we more common in patients with negative BAL. However, injury patterns between patients with positive and negative BAL were similar. One-year mortality was lower among patients with BAL positive. Alcohol use did not have an effect on LOS in ICU. In order to better evaluate the accurate level of alcohol and substance use, screening tests for all trauma patients should be routinely performed. Furthermore, studies with longer follow-up should be done to evaluate whether trauma patients with positive BAL have excessive morbidity or mortality in the long run. As alcohol use seems to be quite common, it should be confirmed that patients receive proper treatment when they exhibit signs of hazardous drinking patterns or alcohol/substance addiction in order to avoid further risks.

## Data Availability

Data sharing not applicable to this article as no datasets were generated or analysed during the current study.

## Abbreviations

AIS: Abbreviated injury scale
BAL: Blood alcohol level
HDU: High dependency unit
ICU: Intensive care unit
ISS: Injury severity score
LOS: Length of stay
MAIS: Maximum abbreviated injury score
NISS: New injury severity score
TAUH: Tampere university hospital
